# A Catalog of Human Genes Associated With Pathozoospermia and Functional Characteristics of These Genes

**DOI:** 10.3389/fgene.2021.662770

**Published:** 2021-07-05

**Authors:** Elena V. Ignatieva, Alexander V. Osadchuk, Maxim A. Kleshchev, Anton G. Bogomolov, Ludmila V. Osadchuk

**Affiliations:** ^1^The Federal Research Center Institute of Cytology and Genetics, Siberian Branch of the Russian Academy of Sciences, Novosibirsk, Russia; ^2^Department of Natural Science, Novosibirsk State University, Novosibirsk, Russia

**Keywords:** male fertility, infertility, spermatogenesis, pathozoospermia, genetic polymorphism, catalog of genes

## Abstract

Genetic causes of the global decline in male fertility are among the hot spots of scientific research in reproductive genetics. The most common way to evaluate male fertility in clinical trials is to determine semen quality. Lower semen quality is very often accompanied by subfertility or infertility, occurs in many diseases and can be caused by many factors, including genetic ones. The following forms of lowered semen quality (pathozoospermia) are known: azoospermia, oligozoospermia, asthenozoospermia, teratozoospermia, and some combined forms. To systematize information about the genetic basis of impaired spermatogenesis, we created a catalog of human genes associated with lowered semen quality (HGAPat) and analyzed their functional characteristics. The catalog comprises data on 126 human genes. Each entry of the catalog describes an association between an allelic variant of the gene and a particular form of lowered semen quality, extracted from the experimental study. Most genes included into the catalog are located on autosomes and are associated with such pathologies as non-obstructive azoospermia, oligozoospermia or asthenozoospermia. Slightly less than half of the included genes (43%) are expressed in the testes in a tissue-specific manner. Functional annotation of genes from the catalog showed that spermatogenic failure can be associated with mutations in genes that control biological processes essential for spermiogenesis (regulating DNA metabolism, cell division, formation of cellular structures, which provide cell movement) as well as with mutations in genes that control cellular responses to unfavorable conditions (stress factors, including oxidative stress and exposure to toxins).

## Introduction

Currently, the global demographic crisis in industrialized countries, including Russia, is accompanied by a decrease in the reproductive potential of human populations. Over the past 60 years, a temporary trend of declining semen quality has been observed, which was expressed as a decrease in sperm counts, increasing prevalence of male infertility and incidence of some urological diseases and congenital abnormalities of the male reproductive system (Joffe, 2010; Levine et al., 2017; Krausz and Riera-Escamilla, 2018).

Understanding all the factors, influencing on male fertility, is of great importance in reproductive medicine for diagnostics and treatment. In clinical practice and population studies, semen analysis is the corner stone of work-up, diagnosis, and treatment of men in the subfertile or infertile couples. The recent WHO laboratory manual for the examination and processing of human semen identified and recommended a set of methods for performing semen analysis (World Health Organization., 2010). The terms used to describe semen quality, as well as the reference values for semen parameters were given in the WHO laboratory manual (World Health Organization., 2010) and are partially presented in Supplementary Table 1 of this paper. Important steps of microscopic investigation of semen include evaluation of sperm concentration, motility, and morphological characteristics. A decrease in the quantitative and/or qualitative semen indicators (one or more) below the reference values is recognized as a lowered semen quality and is most often the cause of male infertility. For example, 755 men suffering from infertility or disorders of the reproductive system were examined at the Moscow Medical and Genetic Research Center, and lowered semen quality was detected in 89% of cases (Andreeva et al., 2017). Lowered semen quality (called pathozoospermia) exists in various forms, including azoospermia, oligozoospermia, asthenozoospermia, teratozoospermia, and some combinations thereof. Azoospermia (absence of spermatozoa in the ejaculate) and severe oligozoospermia (sperm concentration is less than 5 million/ml) represent the most severe forms of male infertility, and men with azoospermia have the highest risk of genetic disorders (25%) (Krausz et al., 2018).

Currently, the following most common genetic causes of male infertility are clearly documented: microdeletions in AZFa, AZFb, and AZFc regions of the AZF locus of the Y chromosome (Kamp et al., 2001; Fernandes et al., 2002, 2006; Krausz et al., 2015; Kim et al., 2017), androgen receptor (*AR*) mutations, including CAG repeat polymorphism in exon 1 (Xiao et al., 2016), chromosomal abnormalities (Alves et al., 2002; Aston and Conrad, 2013; Madureira et al., 2014), cystic fibrosis gene (*CFTR*) polymorphism (Vallières and Elborn, 2014; Jiang et al., 2017). The frequencies of the most common genetic abnormalities associated with male infertility are as follows: chromosomal abnormalities 5–7%, Y-chromosome deletions 5–10%, *CFTR* gene mutations 5%, *AR* mutations 2–3% (Ferlin et al., 2006; Chernykh, 2009). The standard genetic diagnostic examination of infertile men includes karyotype analysis and screening for AZF microdeletions. Karyotype abnormalities and AZF microdeletions are most often detected in men with a sperm concentration less than 5 million/ml (Chernykh, 2009). Screening for *CFTR* mutations in infertile men is carried out after identification of congenital absence of the vas deferens, since mutations in the *CFTR* gene can cause both a severe hereditary disease of cystic fibrosis and aplasia of the vas deferens (Havasi et al., 2010; Ahmad et al., 2013; Jiang et al., 2017). At present, the analysis for the CAG polymorphism of the *AR* gene is not recommended to use in routine diagnostic practice; however, in some cases, it is performed before androgen replacement therapy of patients (Francomano et al., 2013). Moreover, sperm apoptosis markers (Almeida et al., 2005, 2013), DNA fragmentation test (Sá et al., 2015), estimation of sperm DNA methylation (Marques et al., 2004, 2010) and testicular histology (Sousa et al., 2002) are important for male fertility assessment.

The above-mentioned genetic abnormalities contribute significantly to the understanding the nature of male infertility; however, in humans, a set of gene loci associated with reproductive disorders caused by impaired spermatogenesis is much wider. The use of candidate gene approach and some modifications of the whole genome approach (whole-genome and whole-exome sequencing) made it possible to identify other genetic loci (in addition to *AR* and *CFTR* genes, as well as AZF deletions) associated with impaired spermatogenic function (Ferrás et al., 2007; Esteves, 2013; Zhang et al., 2013; Krausz et al., 2015, 2018; Pereira et al., 2015, 2017, 2019; He et al., 2019; Oud et al., 2019; Araujo et al., 2020).

Thus, a large number of genes associated with specific forms of lowered semen quality are currently known. Data on associations are presented both in scientific publications and in non-specialized databases on phenotype-genotype associations: OMIM^1^, ClinVar^2^, HGMD^3^, PheGenI^4^, EGA^5^, GAD^6^ and dbGaP^7^. There is also an Internet portal of the project “Male Fertility Gene Atlas CRU Male Germ Cells” (MFGA)^8^, which accumulates data on genes associated with various disorders of the male reproductive system, including some forms of lowered semen quality. However, as it turned out, each of these databases contains a far from complete set of genes associated with lowered semen quality (Supplementary Table 2). In addition, information may be inaccurate (for example, some genes are incorrectly associated with the trait, or the study of the relationship between the gene and the trait was carried out on a model species (see the comments to Supplementary Table 2). Thus, even with a number of databases on phenotype-genotype associations, additional efforts are needed to extract data on genes associated with specific forms of lowered semen quality and integrate these data, bringing them to a unified format.

The aim of this study was to create a set of genes with polymorphic loci associated with non-syndromic forms of lowered semen quality, as the frequent genetic reason of male infertility. We conducted a search for such genes and their polymorphic loci in scientific publications, and the collected data were presented in the form of a catalog. The functional annotation of the genes from our catalog was then carried out. As a result, we identified a set of biological processes, the genetic control of which can be impaired in individuals with lowered semen quality. The genetic data systematized as a catalog might help the development of genetic panels for the diagnosis of male infertility.

## Materials and Methods

### Extracting Data From Publications

The data was extracted from the publications manually. To find such articles in PubMed^9^ we performed queries to a number of informational resources (databases and information systems). We used search terms referring to various conditions manifested in lowered semen quality (Supplementary Table 1). Four terms (*non-obstructive azoospermia, cryptozoospermia, oligozoospermia, and severe oligozoospermia*) designated conditions associated with a decrease in the number of spermatozoa in the ejaculate. The term *asthenozoospermia* denoted a pathology manifested as an impairment of sperm motility. The terms *teratozoospermia* and its subtype *globozoospermia* denoted conditions characterized by the presence of sperm with abnormal morphology. Since sperm morphological abnormalities are often accompanied by a decrease in sperm motility (Maettner et al., 2014), and both abnormalities in morphology and/or decreased motility can be observed against the background of a decrease in sperm concentration (Guzick et al., 2001; Menkveld et al., 2001), the vocabulary included terms denoting combined forms of lowered semen quality (*asthenoteratozoospermia, oligoasthenozoospermia, oligoteratozoospermia, oligoasthenoteratozoospermia*). The term *azoospermia* was not included in the number of keywords. This was due to the fact that the term *azoospermia* denotes two different conditions: (1) obstructive azoospermia, when spermatogenesis was not disturbed, but an absence of sperm in the ejaculate occurred as a result of mechanical obstruction in genital paths; (2) non-obstructive azoospermia, when an absence of sperm in the ejaculate was due to impaired spermatogenesis, with normal state of the seminal vesicles and vas deferens (Gamidov et al., 2015). Obstructive azoospermia, as a rule, is caused by infections or inflammation of the reproductive tract, trauma and other external factors, and less often by gene mutations (mutations in the *CFTR* gene), and in this case, azoospermia may be present in the clinical picture of the disease as a minor sign. Therefore, only the term *non-obstructive azoospermia* was included in the keywords.

All these forms of lowered semen quality listed in Supplementary Table 1 will hereinafter be referred to as forms of pathozoospermia.

The following databases on phenotype-genotype associations were used as sources of information: (1) OMIM (see text footnote 1); (2) ClinVar (see text footnote 2); (3) HGMD (see text footnote 3); (4) PheGenI (see text footnote 4); (5) EGA (see text footnote 5); (6) dbGaP (see text footnote 7); (7) MFGA (see text footnote 8). We also used ANDSystem^10^ (Ivanisenko et al., 2019) as an additional source of data. ANDSystem contains data on associations between genes and diseases obtained through automatic text-mining analysis of texts collected in PubMed.

### Data Processing

The description of genes was provided with identifiers, official symbols and data on localization on the chromosome obtained from the Entrez Gene database^11^.

Each form of lowered semen quality was assigned to one of three categories (manifestations): (1) low sperm count; (2) reduced sperm motility; (3) abnormal sperm morphology. Combined forms were assigned to two or three categories respectively. The relations between each pathology and an appropriate category (categories) are presented in Supplementary Table 1.

The location of the variant in the gene region (exon, intron, etc.) was extracted from the research paper or from databases (UCSC genome browser)^12^ or dbSNP^13^. Variant genomic location was extracted from dbSNP. If dbSNP rs identifier was not specified in the article, it was identified by queries to databases: (1) to UCSC genome browser if the DNA sequence harboring polymorphic locus was known; (2) to dbSNP or ClinVar^14^ if several variant names (aliases) were found in the research paper (Supplementary Figure 1).

### Web Interface of the Catalog

We used an open-source relational database management system MariaDB 10.2.12 (MariaDB Corporation AB)^15^ to provide access to data from catalog. The web interface was developed with PHP 5.3.3 and it is accessible at https://www.sysbio.ru/hgap/.

### Identification of the Testis-Specific and Testis-Enriched Genes

Tissue-specific genes, that are expressed in the testes, were identified using the TSEA tool (Wells et al., 2015). TSEA tool^16^ uses data on tissue enrichment score of gene expression products (SI) and the corresponding pSI values obtained from the analysis of RNA-seq data across 45 tissue types from the healthy, adult human body (Melé et al., 2015). Depending on the pSI value, calculated for a given tissue, the transcript was considered to be expressed in a tissue-specific manner for this tissue (at pSI < 0.01) or to be expressed in a tissue-enriched manner (at pSI < 0.05).

### Assignment of Genes to Functional Categories

The Database for Annotation, Visualization and Integrated Discovery web-based Functional Annotation Tool (DAVID tool) was applied to the sets of genes from the catalog (Huang et al., 2009). The DAVID tool allowed us to detect the number of genes annotated to each GO term and to identify GO terms that are highly associated with a given gene set (overrepresented GO terms). FDR = 0.05 was used as a threshold criterion characterizing the statistical significance of the excess (enrichment) of the observed number of associations of genes with a specific GO term in comparison with the expected number of associations. The overrepresented GO terms from the Biological Processes vocabulary (GOTERM_BP, GOTERM_BP_5) and Cellular Component vocabulary (GOTERM_CC_5) were considered in our study.

The identification of representative terms characterizing the set of significantly overrepresented GO terms was carried out using the REViGO software^17^ (Supek et al., 2011).

## Results

### The Catalog of Genes Associated With Different Forms of Lowered Semen Quality Caused by Impaired Spermatogenesis (HGAPat): Web Presentation and Information Content

Using the selected terms (Supplementary Table 1) as the keywords and the data collection method described above, we found 126 genes with polymorphic loci associated with various forms of pathozoospermia.

These data are available on the Internet as a relational database. The database consists of four tables: *Genes, Disease, PubGenes*, and *GeneticVariant* (Supplementary Figure 2 shows the database scheme). The *Genes* table contains identifiers, symbols, full names, chromosomal localization, expression pattern and GO annotation of genes. The *Disease* table contains forms of pathozoospermia and their manifestations. The *PubGenes* table contains the name of the gene given in the article, if it differs from the official gene symbol given in Entrez Gene database. The *GeneticVariant* table is the main table that contains data on associations between allelic variants of genes and specific forms of pathozoospermia. So the data are presented in 25 information fields, including 6 identifiers.

The web-interface of HGAPat allows users to observe the collected data and use simple filters: select data by gene, by form of pathozoospermia, by ethnic group, by odds ratio.

**FIGURE 1:**
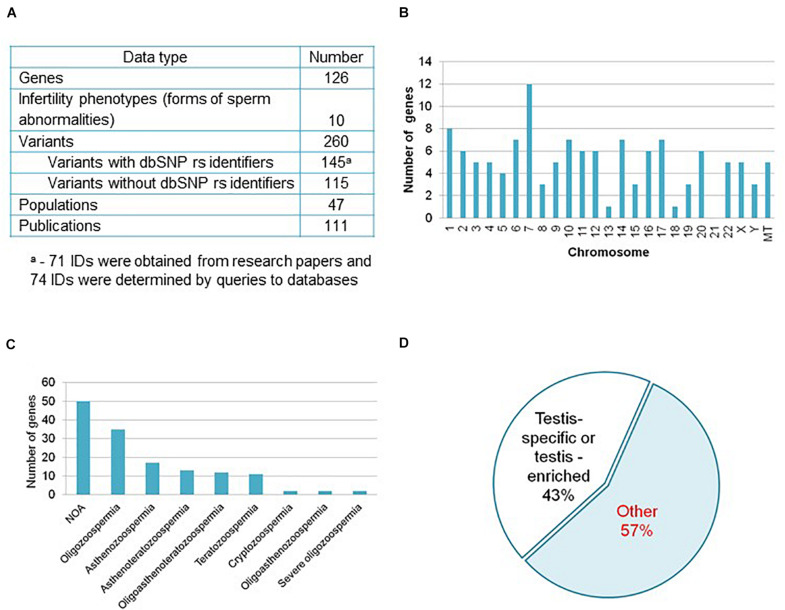
Informational content of the catalog HGAPat. **(A)** HGAPat statistics. Queries to databases were performed according to the scheme presented in Supplementary Figure 1. **(B)** Chromosomal localization of genes. *MT* stands for genes, located in mitochondrial DNA. **(C)** The number of genes associated with a specific form of pathozoospermia (NOA, *non-obstructive azoospermia*). When calculating the number of genes associated with *Teratozoospermia*, genes associated with *Globozoospermia* were taken into account. **(D)** Proportion of genes, expressed in a testis-specific or testis-enriched manner.

Now the catalog HGAPat contains data on 126 genes and 260 variants extracted from 111 publications (Figure 1A). These publications present the results of studies carried out on samples of individuals from 47 different human populations. The largest number of genes was found in Chinese (48 genes) and European (47 genes) populations, as well as in Japanese (11 genes) one.

Most of the genes (89.6% or 113 genes) are located on autosomes, 4.0% and 2.4% (5 and 3 genes) are located on the X and Y-chromosomes, respectively, and 4.0% (5 genes) are located on mitochondrial DNA (Figure 1B). The largest number of genes is associated with such forms of pathozoospermia as non-obstructive azoospermia, oligozoospermia, and asthenozoospermia (Figure 1C).

About sixty percent of the variants (145) described in catalog have dbSNP rs identifiers (Figure 1A) and in half of the cases (74) the identifiers were determined as a result of additional data processing (Supplementary Figure 1). Three quarters of variants are located in exons, 13% are located in introns, 2% are located in UTRs, 2% are located in 5′-near gene or in promoter regions, the rest variants have other location or data on location is not presented.

According to the TSEA tool (see text footnote 16), 43% of genes (54 out of 126) are expressed in the testes in a tissue-specific manner (assigned by the TSEA tool to the testis-specific and testis-enriched categories (Figure 1D and Supplementary Table 4). The remaining 72 genes (57% of the total) are not testis-specific or testis-enriched (in Figure 1D this group of genes is denoted as *Other).*

### Functional Annotation of Genes

The DAVID tool was used to identify biological processes, the genetic control of which may be impaired in patients with pathozoospermia. Using DAVID tool we detected GO terms associated with genes from the catalog more frequently than it was expected by chance. We found the following overrepresented terms from the *Cellular Component* vocabulary: (1) terms denoting the chromosomal localization of proteins (*condensed chromosome, chromosomal part, nuclear chromosome part, etc.*); (2) terms indicating association with the synaptonemal complex (*synaptonemal complex*), cilia (*cilium, motile cilium, ciliary plasm*), axoneme (*axoneme, axonemal dynein complex, dynein complex*), sperm-specific voltage-gated calcium channel (*CatSper complex*) (Supplementary Table 5).

**FIGURE 2:**
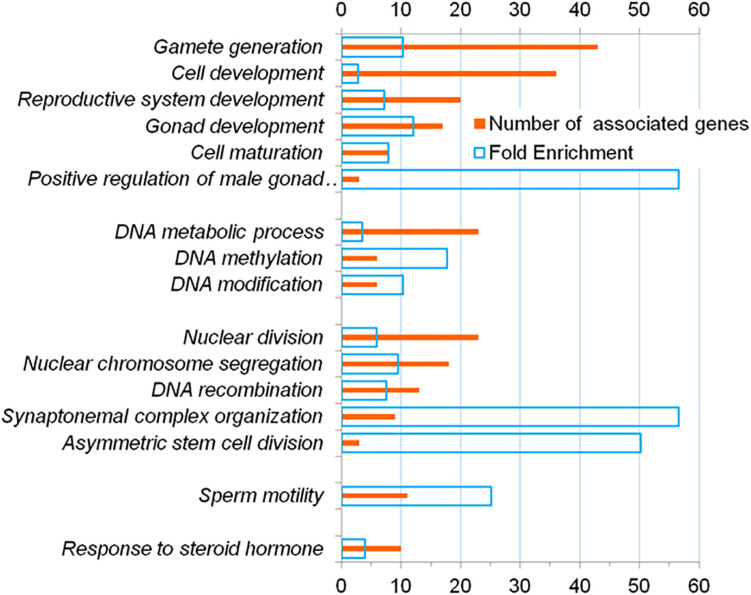
Representative terms from the Gene Ontology database which, according to DAVID, are overrepresented (FDR < 5 * 10^–2^) in the annotations of genes from the catalog HGAPat.

Among the GO terms denoting biological processes, 63 overrepresented (FDR < 0.05) terms were identified (Supplementary Table 6). Using the REViGO program (Supek et al., 2011), we identified sixteen representative terms (Figure 2). These GO terms denoted: (1) development and formation of gametes (*gamete generation, cell development, reproductive system development, gonad development, cell maturation, positive regulation of male gonad development*); (2) metabolic processes associated with DNA (*DNA metabolic process, DNA methylation, DNA modification*); (3) processes associated with cell division (*nuclear division, nuclear chromosome segregation, DNA recombination, saynaptonemal complex organization, asymmetric stem cell division*); (4) motility of the cell (*sperm motility*); (5) hormonal regulation (*response to steroid hormone*).

Then, using the DAVID tool, we analyzed the list of genes from the catalog (72 genes) that were not categorized as testis-specific or testis-enriched (in the Figure 1D these genes were referred to the category *Other*) and found more than seventy over-represented (FDR < 0.05) GO terms denoting biological processes. Among them twenty nine terms were representative according to REViGO (Supplementary Table 7). Most of the representative GO terms found at this step were associated either with the development and formation of cellular organelles (*cellular developmental process, positive regulation of cell differentiation, cellular component assembly, cellular component organization, or biogenesis*, etc.) or with DNA processing (*reciprocal DNA recombination, DNA metabolic process*) or with cell movement (*locomotion, cell motility, localization of cell*). Thus, the set of GO terms identified at this stage specified approximately the same functional characteristics of genes as the set of GO terms identified for the complete list of genes from the catalog (Supplementary Table 6 and Figure 2). In addition, we identified GO terms that denoted the response to unfavorable conditions (*response to oxidative stress, response to oxygen-containing compound, response to stress, response to toxic substance*). These GO terms were associated with 30 genes (in Supplementary Table 7 these genes and GO terms are shown in red).

**FIGURE 3:**
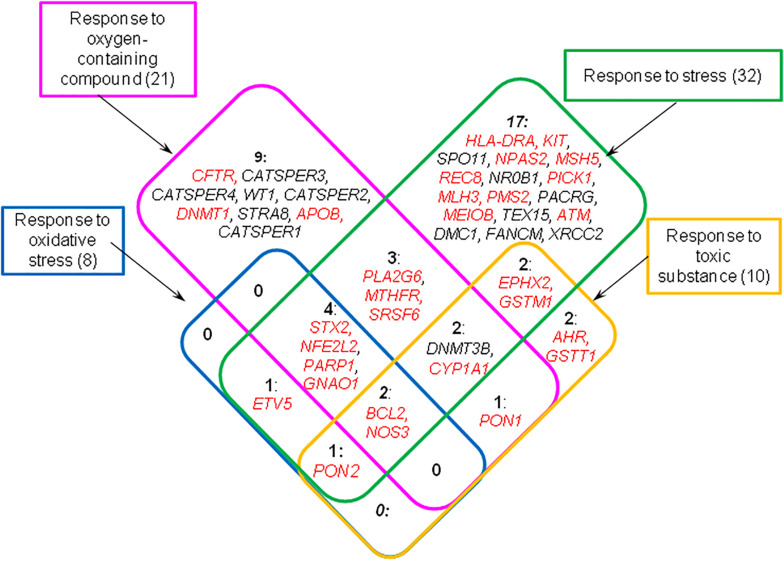
Genes from the catalog HGAPat associated with GO terms designating response to unfavorable conditions (these terms are presented in rectangular boxes). The numbers indicate the number of genes in each group. Genes that are expressed in testis-enriched or testis-specific manner are shown in black; the *other* genes are shown in red.

To compile a complete list of genes related to the response to unfavorable conditions we looked again at the results obtained by DAVID tool for all 126 genes from the catalog and found sets of genes associated with these four GO terms (Supplementary Table 8). In this way we found 44 genes, fourteen of which were testis-specific or testis-enriched (Figure 3).

## Discussion

### The Catalog of Genes Associated With Pathozoospermia

The goal of the current study was to characterize the genetic basis of various non-syndromic forms of pathozoospermia. To achieve this goal, we collected data on genes and their polymorphic loci associated with lowered semen quality caused by impaired spermatogenesis and presented data in the form of a catalog that is publicly available via Internet. Thus, a specialized information resource HGAPat was created that contained more data on the indicated topic than any known open information resource. As an example, Supplementary Table 2 presents the number of genes contained in the catalog and associated with two uncombined forms of pathozoospermia (asthenozoospermia and teratozoospermia), as well as with the considered subtype of azoospermia (non-obstructive azoospermia). The volumes of these data exceed those available in other well-known databases. Likewise HGAPat contains more genes associated with non-syndromic forms of pathozoospermia than the most recent publication (Oud et al., 2019) summarizing knowledge on 521 male infertility genes. We revealed that only 59 genes presented by Oud et al. (2019) (Supplementary Figure 3) met the criteria used in our study (had at least one potentially pathogenic variant described and had relationships with non-syndromic forms of pathologies listed in Supplementary Table 1).

The data we systematized can serve as a basis for the development of prognostic criteria for subfertility and infertility in men based on genetic screening, which is an important but not resolved problem of reproductive medicine.

**FIGURE 4:**
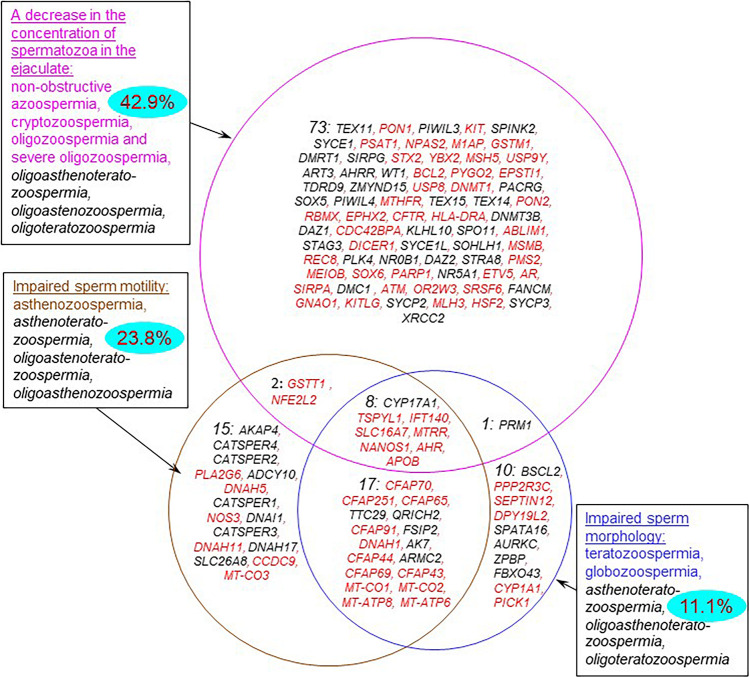
Genes from the catalog associated with various forms of sperm abnormalities. The forms of pathozoospermia are presented in rectangular frames. Combined forms of pathozoospermia are shown in black and *italics*. The numbers in each part of the circles indicate the numbers of genes in each group. The blue ovals show the percentage of genes that control the response to adverse factors (*response to oxidative stress, response to oxygen-containing compound, response to stress, response to toxic substance)*. Genes that are expressed in testis-enriched or testis-specific manner are shown in black; the other genes are shown in red.

Semen abnormalities associated with impaired spermatogenesis (Supplementary Table 1) manifest themselves as: (1) a decrease in sperm concentration; (2) abnormal sperm morphology; and (3) decreased sperm motility. There are the combined forms. In accordance with these manifestations of pathozoospermia, the genes from the catalog can be classified into three groups (Figure 4). The largest number of genes (*n* = 84) was associated with the forms of pathozoospermia associated with a decrease in sperm concentration. Fewer genes were associated with the forms of pathozoospermia due to disturbances in sperm morphology and motility (36 and 42, respectively). Thus, it was found a different degree of genetic heterogeneity of these manifestations of pathozoospermia. Perhaps this can be explained by the different intensity of the study of these manifestations, since the assessment of sperm morphology is a more complex clinical test than the measurement of sperm concentration or the assessment of sperm motility.

Most of genes (77%) were associated with an impairment of one semen parameter (a decrease in sperm concentration, or normal morphology, or motility). At the same time, the groups of genes associated with impaired sperm morphology and motility contained a significant proportion of genes associated with combined forms of pathozoospermia (72% and 64%), especially with asthenoteratozoospermia (25 genes). This finding was in a good agreement with the fact that impaired morphology is one of the possible reasons for a decrease in sperm motility (Maettner et al., 2014).

### Functional Annotation of Genes

The subsequent functional annotation of genes from the catalog allowed us to identify a set of biological processes, the genetic control of which may be disturbed in pathozoospermia.

With the help of DAVID tool, we revealed a tight association of genes from the catalog with a number of biological processes important for the formation of spermatozoa (*DNA recombination, nuclear chromosome segregation, synaptonemal complex organization, sperm motility, etc.* (Supplementary Table 6 and Figure 2) and with the basic cellular structures of spermatozoa (*condensed chromosome, synaptonemal complex, motile cilium* etc.; Supplementary Table 5). The results obtained are in good agreement with the idea that the cellular processes mentioned above (DNA replication, chromosome segregation, formation of a synaptonemal complex) are essential for the formation of a mature sperm cell (Hann et al., 2011; Fowler et al., 2019) and at the same time a complex cellular apparatus is formed that ensures the movement of spermatozoa (O’Donnell, 2014; Pereira et al., 2017; Cannarella et al., 2020). In particular, proteins involved to synaptonemal complex assembly are crucial for spermatogenesis (Wellard et al., 2020). It was shown that infertile men were characterized by increased levels of sperm aneuploidy likely due to increased errors in meiotic recombination and chromosome synapsis (Tempest, 2011).

We found that only 43% of genes are expressed in a testis-specific or testis-enriched manner (Figure 1D and Supplementary Table 4), and the rest of genes do not belong to this category. Our data are in agreement with those of the other researchers who showed that only 933 of 3,580 genes differentially expressed in the testes of infertile and fertile men were undetectable in 45 embryonic and adult non-testicular tissues (Chalmel et al., 2012). The results obtained confirm the idea that spermatogenesis is closely related with health of the whole organism (Omu, 2013).

In order to identify the most significant biological processes associated with the rest part of genes (which we categorized as *others*, Figure 1D), we performed the additional analysis using the DAVID tool. We found 29 representative biological processes associated with genes from this group (Supplementary Table 7). Many of the processes found at this step were directly related to the formation of spermatozoa and their cellular functions (*cellular developmental process, locomotion, cell motility, DNA metabolic process*), which was in good agreement with the results obtained for the complete list of genes. Along with this, we identified a group of 44 genes (one third of the total number of genes) associated with the response to adverse environmental factors (Figure 4 and Supplementary Table 8). Among them there were the genes encoding proteins, the protective role of which is very well studied. For example, *GSTM1, GSTT1, CYP1A1*, and *EPHX2* encode xenobiotic-metabolizing enzymes (Pande et al., 2008; Decker et al., 2009). *AHR* encodes ligand-activated transcription factor involved in the regulation of biological responses to aromatic hydrocarbons and oxidative stress (Dietrich, 2016). *PON1* and *PON2* encode enzymes that modulate oxidative stress and inflammation (Furlong et al., 2016).

The identification of such a group of genes indicates that the genes, which control the response to adverse factors, can play an important role in maintaining male reproductive function, namely, in maintaining adequate sperm quality. The largest fraction of such genes (42.9%) was found in the set of genes associated with a low sperm count (Figure 4). Smaller fractions were found among the sets of genes associated with reduced sperm motility and abnormal morphology (23.8 and 11.1%, respectively). However, the identification of genes controlling the response to unfavorable conditions among the genes of all three groups suggests that three manifestations of pathozoospermia (a decrease in sperm count, abnormal sperm morphology and motility) may be associated with unfavorable environmental conditions. Negative external factors including environmental pollution, smoking, oxidative stress etc. are known to affect male fertility, resulting in decreasing sperm count, sperm motility and percentage of morphologically normal sperm as well as sperm DNA damage (Omu, 2013; Lafuente et al., 2016). Our results are in agreement with these evidences, demonstrating that genetically determined sustainability to environmental factors is crucial to male fertility.

## Data Availability Statement

The original contributions presented in the study are included in the article/Supplementary Material, further inquiries can be directed to the corresponding author/s.

## Author Contributions

EI performed search for publications and manual extraction of data from the articles, processed data, created the general scheme of the catalog HGAPat, interpreted the data, and wrote the manuscript. AO interpreted the data and corrected the manuscript. MK performed manual extraction of data on genetic variants from the articles and interpreted the data. AB developed the software, wrote, and corrected the manuscript. LO provided overall supervision of the project, interpreted the data, and corrected the manuscript. All authors read and approved submission of this manuscript.

## Conflict of Interest

The authors declare that the research was conducted in the absence of any commercial or financial relationships that could be construed as a potential conflict of interest.

## Footnotes

1 https://www.ncbi.nlm.nih.gov/omim

2 https://www.clinicalgenome.org/data-sharing/clinvar/

3 http://www.hgmd.cf.ac.uk/ac/index.php

4 https://www.ncbi.nlm.nih.gov/gap/phegeni

5 https://ega-archive.org/

6 https://geneticassociationdb.nih.gov/

7 https://www.ncbi.nlm.nih.gov/gap/

8 https://mfga.uni-muenster.de/projectInfo.html

9 https://pubmed.ncbi.nlm.nih.gov/

10 http://www-bionet.sscc.ru/and/cell/

11 https://www.ncbi.nlm.nih.gov/gene

12 https://genome.ucsc.edu/

13 https://www.ncbi.nlm.nih.gov/snp/

14 https://www.ncbi.nlm.nih.gov/clinvar/

15 https://mariadb.org/

16 http://genetics.wustl.edu/jdlab/tsea/

17 http://revigo.irb.hr/
