# Supplementary material for: A Catalog of Human Genes Associated With Pathozoospermia and Functional Characteristics of These Genes
Source: Front Genet. 2021 Jul 5;12:662770. doi: 10.3389/fgene.2021.662770 (PMC8287579; doi:10.3389/fgene.2021.662770)
Supplement: Supplementary file 2 [file Data_Sheet_2.docx]

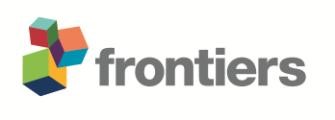


***Supplementary Material***


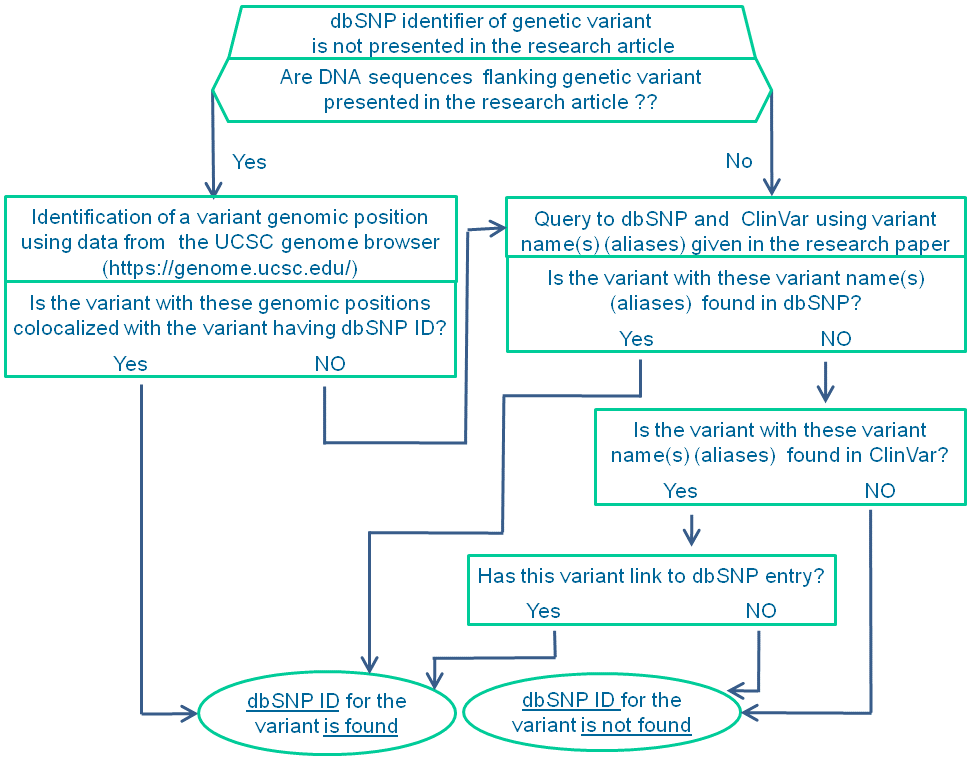


**Supplementary Figure 1.** Search for dbSNP identifiers for those genetic variants for which dbSNP identifiers were not indicated in the research articles.

Supplementary Material


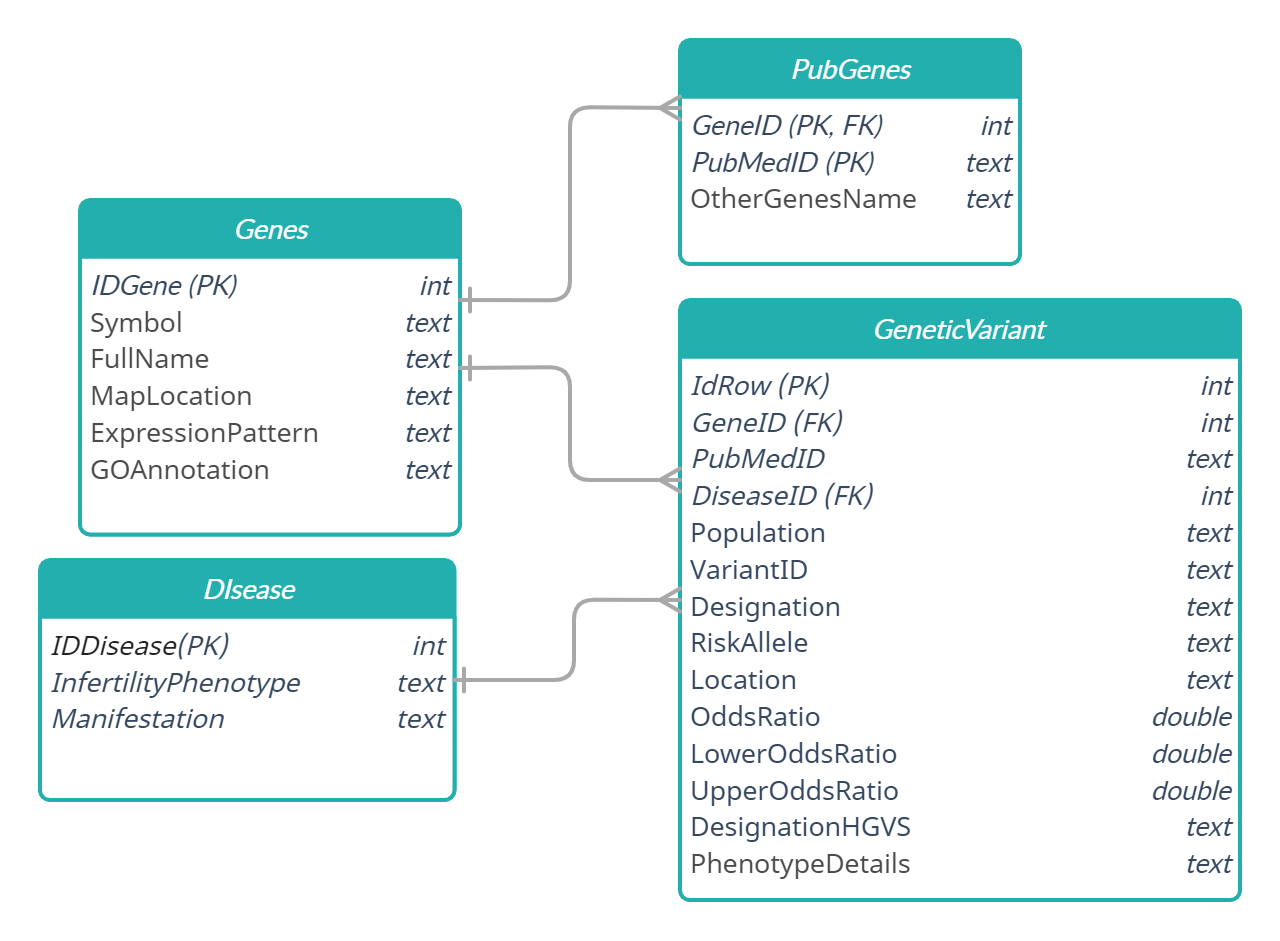


**Supplementary Figure 2.** General scheme of the сatalog (ER-model of the database). The primary and foreign keys are marked PK and FK, accordingly.

2


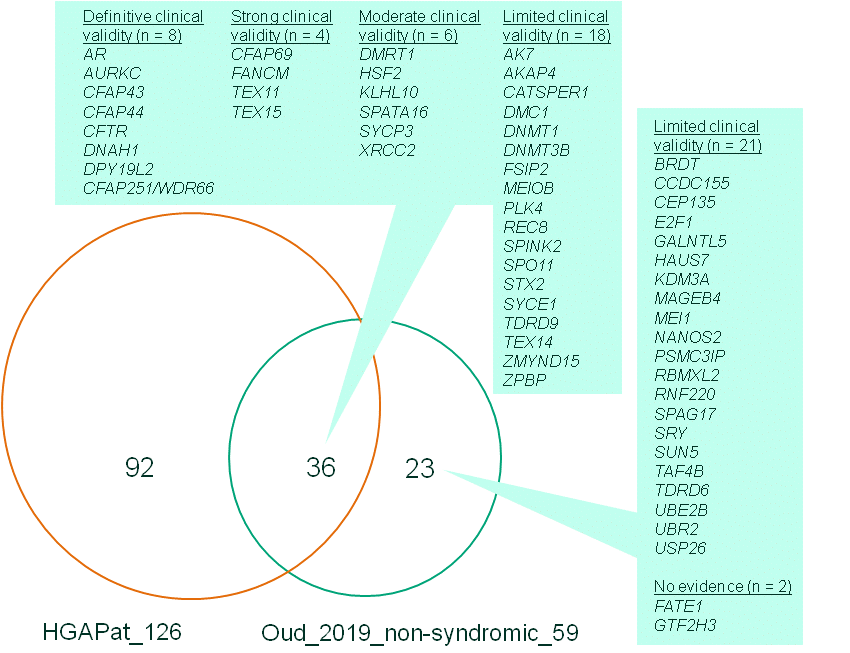


**Supplementary Figure 3.** Intersection between two lists of genes. *HGAPat_126* designates all 126 genes from the catalog HGAPat presented in the current report. *Oud_2019_non-syndromic_59* designates the list of 59 genes from [Oud et al. 2019] that have at least one potentially pathogenic variant described and had relationships with non-syndromic forms of pathologies manifested in decreased quality of semen (pathologies are listed in Table S1). These 59 genes were extracted from the TableSIV published by [Oud et al. 2019]. This figure demonstrates that (1) HGAPat contains more genes that meet the above criteria; (2) according to [Oud et al. 2019], genes that were not found in HGAPat, but were present in the [Oud et al. 2019], have low clinical validity.

**References**

1. Oud, M.S., Volozonoka, L., Smits, R.M., Vissers, L.E., Ramos, L., Veltman, J.A. (2019). A systematic review and standardized clinical validity assessment of male infertility genes. Human Reproduction. 34(5), 932-941. doi: 10.1093/humrep/dez022.

3
